# Insights on suicide postvention for healthcare institutions in the Philippines: a health systems framework approach

**DOI:** 10.3389/fpsyg.2025.1735407

**Published:** 2025-11-26

**Authors:** Evangeline Bascara dela Fuente, Rowalt Alibudbud

**Affiliations:** 1 Department of Psychiatry and Behavioral Medicine, College of Medicine, University of the Philippines – Manila, Manila City, Philippines; 2 National Teachers Training Center for the Health Professions, University of the Philippines – Manila, Manila City, Philippines; 3 Department of Sociology and Behavioral Sciences, College of Liberal Arts, De La Salle University, Manila City, Philippines

**Keywords:** suicide postvention, mental health, suicide prevention, Philippines, hospitals, healthcare institution, suicide

## 1 Introduction

According to the WHO (2025), more than 700,000 people die by suicide every year. Each of these deaths can leave behind an intricate web of grief, guilt, and unanswered questions that reach far beyond the individual, affecting families, communities, and the institutions in which that person lived or worked (Tal Young et al., 2012). Globally, there has been growing attention to suicide prevention, yet the parallel concept of suicide postvention remains overlooked, especially in low- and middle-income countries like the Philippines (Redaniel et al., 2011; WHO, 2025). Postvention aims to support those left behind who may bear the emotional impact of the loss and seeks to reduce the risk of adverse sequelae such as further suicides (Abbate et al., 2024).

Research from high-income countries has shown that healthcare professionals are at an increased risk of suicide compared with the general population. In the Philippines, the issue of suicide in healthcare settings remains both sensitive and underexamined (Lagman et al., 2021; Redaniel et al., 2011). Healthcare institutions are places designed to preserve life. When suicide enters those walls, it can unsettle the very identity of care itself (Horowitz et al., 2020; Sakinofsky, 2014). When suicide occurs within an institution, responses are often improvised and focused on immediate containment rather than long-term recovery or reflection (Ballard et al., 2008). Healthcare workers may return to work quickly, haunted by unanswered questions, while leadership struggles to navigate the emotional and reputational aftermath.

The absence of structured postvention mechanisms may not merely represent an administrative gap, but instead signal a broader neglect of emotional recovery within health institutions. There is a need for systemic interventions, such as structured postvention protocols, to enhance resilience, foster open dialogue, and normalize psychological care within institutions.

## 2 Objectives

This opinion piece examines established frameworks and literature to guide suicide postvention in healthcare settings following the loss of a colleague or patient. In the Philippines, where healthcare professionals serve under immense strain, suicide postvention offers a path to healing and resilience, honoring both patients and caregivers. It helps institutions address caregiving's emotional toll, affirm healthcare workers' dignity, and turn grief into collective learning.

## 3 Discussion

### 3.1 Suicide in the healthcare sector of the Philippines

Suicide, once rarely discussed in public, has become a growing concern, especially among young adults and students (Lagman et al., 2021; Malolos et al., 2021; Martinez et al., 2020). Nonetheless, growing concerns emphasize the importance of postvention in the Philippines, with the World Health Organization (n.d.) placing the 2019 suicide rate at roughly 3.42 per 100,000. While the country's rate is lower than the 9.1 deaths per 100,000 globally WHO, (2024), suicide is a silent but deepening concern within the healthcare workforce (Awan et al., 2022). Although comprehensive national data are scarce in the Philippines, anecdotal accounts and small-scale reports point to suicide incidents among medical students, nurses, and resident physicians over the years (Galicia and Bautista, 2018; Tan, 2017).

The culture of healthcare in the country, like in many parts of the world, emphasizes endurance, professional detachment, and self-sacrifice (Alibudbud, 2022; Borbolla and Nkansa-Dwamena, 2025). While these values have helped Filipino healthcare workers sustain compassion amidst heavy workloads and resource constraints, they leave little room for vulnerability and contribute to a quiet but pervasive crisis of burnout, exhaustion, and despair (Alibudbud, 2023a,b; Lagman et al., 2021; Redaniel et al., 2011). The emotional and physical demands of healthcare work, coupled with its hierarchical culture, create an environment where distress can easily go unnoticed. Long shifts, exposure to suffering, moral distress, and limited access to confidential support contribute to chronic stress (Alibudbud, 2023a; Carandang et al., 2024; Koinis et al., 2015). Staff mental health can remain a delicate topic, often overshadowed by service demands and professionalism (Gunasekaran et al., 2022).

However, the same cultural norms that promote unity can also inhibit open emotional expression (Martinez et al., 2020). Likewise, hierarchical relationships may discourage staff, especially junior staff, from voicing their concerns that contributed to the incident (Lee and Lee, 2024). Cultural stigma further complicates help-seeking. Mental illness is often interpreted through moral, religious, or familial lenses, and shame can deter individuals from disclosing distress (Martinez et al., 2020).

When a suicide occurs within a healthcare institution, its aftermath ripples through teams and wards (Ballard et al., 2008). The implications extend far beyond the immediate loss. Each incident exposes the fragility of the systems meant to protect both patients and caregivers (Abbate et al., 2024; Evans et al., 2023). Healthcare workers may struggle with guilt, self-blame, or anger. Supervisors, meanwhile, may face moral injury or administrative scrutiny (Ballard et al., 2008).

The absence of postvention mechanisms can lead to compassion fatigue, absenteeism, and disengagement, undermining both workforce morale and patient safety (Abbate et al., 2024; Evans et al., 2023). From a public health standpoint, an uncoordinated response may contribute to further self-harm among those closely affected. The lack of structured support can erode trust, impair teamwork, and perpetuate a culture of silence (Abbate et al., 2024; Evans et al., 2023).

Within the tragedy lies an opportunity for institutional learning. Postvention must be recognized as an integral component of healthcare governance and not an optional crisis response. It enables organizations to identify systemic contributors, such as overwhelming workloads, unclear communication channels, and inadequate mental health resources, and to strengthen their resilience mechanisms (Abbate et al., 2024; Evans et al., 2023). A framework fit for purpose must necessarily consider the nature of mental health, the peculiarities of healthcare institutions, and the sociocultural context in the Philippines.

### 3.2 Suicide postvention in the Philippine setting utilizing the WHO Health System Framework

In the Philippines, responses to suicide cannot be divorced from the nation's cultural fabric (Martinez et al., 2020; Redaniel et al., 2011). Filipino society is deeply relational, grounded in the values of *pakikipagkapwa* (shared humanity), *bayanihan* (collective support), and *malasakit* (empathetic concern). Filipinos possess strong coping mechanisms rooted in their family and spirituality (Martinez et al., 2020). These values, when consciously integrated into institutional policies, can transform how healthcare organizations respond to tragedy (Bersamira and Macaraeg, 2022; Martinez et al., 2020; Rogayan and Macalinao, 2024).

The WHO Health System Framework can guide the development of a comprehensive suicide postvention approach in healthcare settings (WHO, 2007). This framework identifies six key building blocks of a functioning health system, including leadership and governance, service delivery, health workforce, information systems, access to essential medicines, and financing (WHO, 2007). Applied to postvention in the Philippine context, these components can guide the design of coordinated, sustainable responses that extend beyond crisis management toward a culture of wellbeing and institutional resilience.

First, institutional leaders must exhibit *malasakit*, including responding to loss with empathy, transparency, and vulnerability (Bersamira and Macaraeg, 2022; Martinez et al., 2020; Gamad et al., 2025). Compassionate leadership must be a cornerstone of institutional healing through the creation of a comprehensive postvention program (de Zulueta, 2015). Such programs have legislative support, as the 2018 Mental Health Act mandated accessible, community-based services and workplace mental health programs (Alibudbud, 2023b; Maravilla and Tan, 2021). Administrators and unit heads should be equipped to effectively manage suicide incidents. Establishing postvention committees that can be activated as needed is an important step. At its most basic, such committees include an administrator, a mental health provider, a human resource coordinator, a communication expert, and a spiritual consultant (Dyrbye et al., 2020). Postvention should be integrated into quality assurance and patient safety systems, rather than being treated as a separate activity.

Second, a postvention program must recognize the relevance of the biopsychosocial model in understanding and responding to mental health concerns, the nature of mental health and illness, and the merit of a staged approach to addressing mental health concerns in contrast to the binary approach commonly employed (Bascara-dela Fuente et al., 2025; Patel et al., 2018).

The program includes immediate crisis management, support for affected teams and families, and long-term monitoring of staff wellbeing (Abbate et al., 2024; Evans et al., 2023). Peer-support programs, confidential counseling, and structured return-to-work plans after traumatic events can help normalize care-seeking and prevent burnout (Alibudbud, 2023a; Simms et al., 2023).

A culturally grounded postvention model involves providing psychological support that respects *hiya* (a sense of modesty and privacy), ensuring confidentiality and cultural sensitivity (Martinez et al., 2020; Rogayan and Macalinao, 2024). Collective mourning can be facilitated through prayer vigils, memorial masses, or silent reflection (Bersamira and Macaraeg, 2022; Martinez et al., 2020; Gamad et al., 2025). In coordination with mental health professionals, activities such as prayer groups, family-inclusive counseling, or remembrance events can foster a sense of belonging and resilience. These activities acknowledge the spiritual dimension of grief and reaffirm communal bonds. *Bayanihan* can be promoted through formal peer mentorship and psychological first aid programs that support colleagues as equals (Bersamira and Macaraeg, 2022). The term “postvention” may sound foreign or too clinical, so alternatives like “*pakikipagkapwa”* or “wellness response” may resonate more deeply with Filipino sensibilities.

Third, access to psychotropic medications and other forms of biological treatments ensures that the need to address the biological aspect of mental health and illness is addressed in keeping with the biopsychosocial model (Melhem et al., 2023).

Fourth, human resource needs must be addressed. Mental health has historically been underfunded in the Philippines, with only 5% of the total public health expenditure allocated to it (Alibudbud, 2023b; Maravilla and Tan, 2021). Human resource shortages continue to undermine service delivery (Alibudbud, 2023b, 2024). Interprofessional collaboration is recommended as a means to resourcefully meet the need for warm bodies in the various services that must be provided. Aside from mental health specialists, there is merit in partnering with non-specialists (WHO, 2010; Bascara-dela Fuente, 2025). Partnering with chaplains, peer facilitators, and family representatives to conduct memorials that emphasize collective healing rather than blame.

Fifth, confidential data collection on suicide cases, contributing factors, and follow-up outcomes is vital. Such information enables learning, risk mapping, and the identification of institutional vulnerabilities (Schatten et al., 2020). institutionalizing “learning from loss” sessions to identify systemic pressures that may have contributed to distress, promoting accountability without stigmatization.

Sixth, sustainable postvention can require designated funding. Hospitals should allocate a portion of operational budgets to mental health services, including staff wellness and crisis response training.

Overall, by operationalizing postvention across these six domains, healthcare institutions can evolve from reactive crisis management to proactive mental health stewardship. Ultimately, a Filipino-informed postvention framework blends evidence-based mental health practices with cultural wisdom. It recognizes that healing is both personal and communal and that the journey from grief to resilience can honor both professional ethics and cultural identity.

## 4 Conclusion

The institutionalization of suicide postvention in Philippine healthcare requires a strong policy backbone. Government authorities like the Department of Health, in partnership with professional regulatory bodies, can spearhead the development of national postvention guidelines aligned with the Mental Health Act. These should define institutional roles, confidentiality standards, and mechanisms for reporting and evaluation. Accreditation systems, such as those used by state health insurance like PhilHealth or hospital licensing bodies, could incorporate postvention readiness as a quality indicator, encouraging hospitals to establish dedicated programs.

At the organizational level, healthcare facilities can establish multidisciplinary postvention teams, comprising clinicians, counselors, chaplains, human resources personnel, and administrators. These teams would coordinate immediate crisis responses, conduct psychological first aid, and ensure long-term monitoring of affected individuals. Regular simulation exercises, reflection sessions, and leadership workshops can help sustain readiness and empathy. Integrating postvention into employee wellness initiatives ensures continuity, while partnerships with universities, mental health NGOs, and professional associations can expand expertise and support networks.

Despite its urgency, suicide postvention remains an under-researched field in the Philippines (Alibudbud, 2025; Lagman et al., 2021; Redaniel et al., 2011). Thus, future studies can explore suicide prevalence among healthcare workers, examine institutional responses, and evaluate the cultural adaptability of existing models. Mixed-method research combining quantitative data and narrative inquiry can shed light on lived experiences, including how healthcare professionals grieve, recover, and rebuild after a colleague's suicide. Implementation science can also help identify barriers and facilitators to sustaining postvention programs over time.
